# Good and Bad Teeth

**Published:** 1869-05

**Authors:** 


### ARTICLE VII.

### *Good and Bad Teeth.*

In one of the scientific almanacs of Great Britain for this year, we find the following article.

The best advice to those who wish to preserve a sound set of teeth to old age, (a most important aid to general health) is that they should be carefully brushed and thoroughly cleaned *after* every meal, and particularly on going to bed at night—surely a far more reasonable practice than rising, when the mouth should be fresh and sweet in a healthy state, a luxury which we fancy is but seldom enjoyed by the smoking and grog drinking gentry of the present day.

Still, either for want of care, or perhaps sometimes in spite of care, toothach is to great numbers of people one of the most vexatious among the minor scourges of life, and no disease of a simple nature so completely mocks the efforts of the profession and the quacks, and baffles all their vaunted remedies and specifics. The art of dentistry has of late made an immense stride by the general introduction of nitrous oxide as an anæsthetic. That this will prove successful in relieving the hitherto frightful pain of tooth extraction is, we believe, almost beyond a doubt, and there seems every reason to believe that it will prove a safe application as well as an effectual one. Extraction, however, is not an advisable course to pursue, except those which are, of course, better out than in, and it is often practiced in cases which render it completely absurd. To our damp and variable climate is doubtless due much of the prevalent neuralgia of the country, and a cold in the face, to which some people are especially liable, is also to be traced to the same cause. The homœopathists, we believe, have had considerable success in the treatment of that form of the latter which is characterized by soreness and inflammation, by the administration of mercurius vivus, of course in dilution. Of this, however, we know but little, but we are very confident of the value, in cases of every kind, of the following dose in two pills, at least, as preparatory to the cure:

| | | |
|---|---|---|
| ℞. | Pil. hydrag., | gr. v. |
| | Pil. rhei co., | gr. v. |
| | Ext. belladonnæ, | gr. 1-8. |

In cases of colds in the face, the old fashioned application of a "pepper plaster" has almost invariably a soothing effect, though its success is unfortunately attended with but little profit to the druggist. It consists merely of a piece of brown paper almost as large as the cheek, soaked in vinegar and well sprinkled with pepper, secured over the face during the night.

Neuralgia is to some extent independent of the teeth, though where these are perfect, its visits are not near so probable, nor so frequent. There are two forms of this wearying complaint, which are totally distinct, and yet which are frequently confounded. One, and this is we are convinced by far the most common, is the pain resulting from some trifling cause, or perhaps directly traceable to none, when the nervous system is in a state of more or less complete prostration, and when the manifestations of this debility are chiefly in the facial extremities. In cases of this kind *opium* and all its preparation should be most carefully avoided, and a purely tonic treatment is required. Quinine is not by any means the best tonic we possess, and in these cases is far inferior to iron—especially the sulphate. But in neuralgia proper—that is, in its intemittent form—quinine unquestionbly ranks first, not, be it understood, from its tonic virtue, which is, as we have said, inferior to that of many other medicines, but from its special property as an anti-intermittent. In such cases as these, indeed, pure tonics—iron, for example —have little or no effect, while next to quinine, arsenic is perhaps the best remedy, and this has no tonic value whatever. One or two drops of Fowler's solution given alternately with one or two grains of quinine, at rather frequent intervals, is often very efficacious, though we need not say that the utmost care is necessary to guard against an over-dose of such a poison as arsenic.

We have made these remarks because we regard the treatment of toothache and neuralgic affections as a tolerably fair portion of a druggists practice at the counter. It is generally a most extensive portion, and we need hardly say that a man who can acquire the reputation of being able to cure the toothache is a man who must command esteem. The experience of an intelligent chemist will be sure to have led him to know much about these complaints, but it may be that the hints we have thrown out above may add an idea to some who wish to be as successful as possible even in their transgressions of etiquette, and while the writer deprecates most strongly the unauthorized assumption of general duties by those who have not dared, or at least, have not chosen to submit their abilities to a fair test, such as is prescribed by the law of the land, but have relied on their own natural "unerring instinct" in the treatment of all sorts of diseases, he is still slightly tempted to regard these minor complaints somewhat as the public does—viz., fairly in the province of the chemist and druggist.

In addition to the above remarks, we have been favored with the following, from a practical dentist of great experience, and, though his opinions somewhat conflict with our own in one or two instances, we gladly insert them for the sake of their presumptive value. First of all a very important point to remember is that the teeth should always be cleaned with warm or tepid water, or, better still, with a cool tea, the tannin of which exerts its astringent properties on the gum. The *rationale* of this observation is that the enamel, being mechanically brittle and usually warm, the sudden application of cold water to this is very liable to cause it to crack—a condition which is very frequently met with.

Real cases of neuralgia are much more rare that is generally supposed. A disease which is frequently mistaken for it is what is technically known as *exostosis*—that is, a particular granular enlargement of the fangs. This state is usually met with about the spring and fall of the year, and is the occasion of the violent pains in the nerve-centres, shooting from the temples to the neck, which are generally, like neuralgia proper, intermittent in their attack. Sound and decayed teeth are equally liable to this condition, and nothing but extraction or patience can be of any effectual aid in removing such a disorder.

Inflammation of the periosteum is one of the most general causes of toothache, and is a usual consequence of cold. The periosteum is the fine membrane covering the fangs of the teeth, is highly organized, and is, therefore, exceedingly sensitive. To abate this inflammation a small dose of mercurial pill, with not more than the eighth of a grain of extract of belladonna, should be given night and morning. Warm fomentations ought to be frequently applied, and every care taken to prevent the further influence of cold. In many cases a leech on the gum will very readily relieve this form of toothache.

Speaking very exactly, toothache is an inflamed state of the central nerve of the tooth. As a rule, it may be considered impossible for this nerve, or the pulp of the tooth—that is, its main portion—to become affected in this manner until after the enamel has been partially or entirely removed. The best application in such cases is, after thoroughly cleansing the hollow with a small piece of cotton, and afterwards disinfecting with creosote, to plug with a pellet of wool soaked in a saturated solution of gum benzoin and tannin in chloroform. The well known Bunter's Nervine appears to be something similar to this solution.

In the management of the teeth, too great prominence cannot be given to perfect cleanliness. All foreign particles should be carefully removed after meals by the use of a tooth brush, or where this is not sufficient, a tooth-pick, made of soft wood, is admissible; but those formed of metallic or other hard substances should be avoided. A saponaceous tooth-powder is the most serviceable, if any be employed. The dentist should be as conservative as possible; extraction is seldom necessary if he be skilled in his art; exostosis and abscess at the fang being, however, diseases which imperatively require this to be resorted to.—*Med. & Surgical Reporter.*

---

